# Long-term outcomes of laparoscopic Extralevator Abdominoperineal excision with modified position change for low rectal Cancer treatment

**DOI:** 10.1186/s12885-022-10019-2

**Published:** 2022-08-24

**Authors:** Shaowei Sun, Shengbo Sun, Xiangyun Zheng, Jiangtao Yu, Wenchang Wang, Qing Gong, Guowei Zhao, Jing Li, Huanhu Zhang

**Affiliations:** Department of Gastrointestinal Surgery, Weihai Municipal Hospital, Cheeloo College of Medicine, Shandong University, Weihai, 264200 Shandong Province China

**Keywords:** Low rectal cancer, Exralevator abdominoperineal resection, Position change, Long-term, Survival analysis

## Abstract

**Background:**

Extralevator abdominoperineal excision (ELAPE) has been recommended for treating low rectal cancer due to its potential advantages in improving surgical safety and oncologic outcomes as compared to conventional abdominoperineal excision (APE). In ELAPE, however, whether the benefits of intraoperative position change to a prone jackknife position outweighs the associated risks remains controversial. This study is to introduce a modified position change in laparoscopic ELAPE and evaluate its feasibility, safety and the long-term therapeutic outcomes.

**Methods:**

Medical records of 56 consecutive patients with low rectal cancer underwent laparoscopic ELAPE from November 2013 to September 2016 were retrospectively studied. In the operation, a perineal dissection in prone jackknife position was firstly performed and the laparoscopic procedure was then conducted in supine position. Patient characteristics, intraoperative and postoperative outcomes, pathologic and 5-year oncologic outcomes were analyzed.

**Results:**

The mean operation time was 213.5 ± 29.4 min and the mean intraoperative blood loss was 152.7 ± 125.2 ml. All the tumors were totally resected, without intraoperative perforation, conversion to open surgery, postoperative 30-day death, and perioperative complications. All the patients achieved pelvic peritoneum reconstruction without the usage of biological mesh. During the follow-up period, perineal hernia was observed in 1 patient, impaired sexual function in 1 patient, and parastomal hernias in 3 patients. The local recurrence rate was 1.9% and distant metastasis was noted in 12 patients. The 5-year overall survival rate was 76.4% and the 5-year disease-free survival rate was 70.9%.

**Conclusions:**

Laparoscopic ELAPE with modified position change is a simplified, safe and feasible procedure with favorable outcomes. The pelvic peritoneum can be directly closed by the laparoscopic approach without the application of biological mesh.

**Supplementary Information:**

The online version contains supplementary material available at 10.1186/s12885-022-10019-2.

## Background

In recent years, increasing evidence suggest higher rates of positive circumferential resection margin (CRM), intraoperative perforation, and local recurrence in Abdominoperineal resection (APE) as compared with anterior resection (AR) [1–3]. Positive CRM and intraoperative perforation are closely related to the local recurrence of rectal cancer, which compromises oncological outcomes [4–6]. The mesorectum narrowed gradually from top to bottom, and disappeared at the plane of puborectal ring. Consequently, when separating this place, the muscular layer of the bowel wall is easily to be cut and intestinal perforation occurs frequently. Furthermore, low rectal tumors usually locate in the exposed area of the mesorectum [7]. As a consequence, when performing an APE, the surgical specimens usually existed a narrow segment in here, which is called as “Morson waist”. Evidence have indicated that positive CRM and intraoperative perforation were closely related to the Morson waist [6]. Hence, traditional APE conducted in combination with TME theory does not lower the intraoperative perforation incidence and CRM positive rate [2, 8].

Demand of reducing the incidence of positive CRM and intraoperative perforation led to the emergence of extralevator abdominoperineal excision (ELAPE) [9]. In ELAPE, the levator muscles were totally excised to form a cylindrically shaped specimen and the Morson waist could be avoided. Increasing clinical evidence disclosed the oncology superiority of ELAPE over the conventional APE [10, 11]. In ELAPE, the abdominal procedure is firstly conducted as with standard APE in lithotomy position, and the extralevator excision is then performed via perineal approach with the requirement of position change from the lithotomy position to the prone jackknife position. However, the intraoperative position change increases the risk of prolonging operation time. As a result, whether the benefits of intraoperative position change to a prone jackknife position outweighs the associated risks remains controversial. A previous study on laparoscopic ELAPE conducted by Keller et al. [12] showed that perineal approach in prone jackknife position was not an essential condition to complete levator muscle resection in perineal operation. Successful levator muscle resection could be achieved in lithotomy position in laparoscopic approach. Studies by Chi et al. [13] and Zhang et al. [14] also supported that the resection of levator muscle in lithotomy position was feasible in laparoscopic approach. Nevertheless, evidence from Xiao et al. [15] indicated that the short-term outcomes of laparoscopic ELAPE without intraoperative position change were similar to that of the conventional APE. Moreover, a large multi-center study suggested that perineal dissection in prone jackknife position is an independent factor contributing to reducing the occurrence rates of positive CRM and intraoperative perforation [10]. Another study reported short-term outcomes of ELAPE in prone position showed a low CRM positive rate [16].

In addition to the above controversy, the position change sequence of this surgical technique confers several potential disadvantages. First, in the process of reversing patient’s position and in the prone jackknife position, the abdomen and colostomy stoma would inevitably be squeezed, which increase the possibilities of abdominal incision disruption, incisional hernia, avascular necrosis and dysfunction of colostomy stoma, and even short-term complications, especially in patients with obesity and abdominal wall weakness [9, 17]. Second, errhysis caused by the abdominal procedure in ELAPE would seriously influence the operative field of the perineal procedure and hence increase patient risks, particularly in situations when tumor infiltrates to prostate, vesicula seminalis, vagina, cervix and coccyx [9]. Third, ELAPE requires to disarticulate the coccyx from the sacrum [9], which increases the risks of trauma and postoperative sacrococcygeal pain. Moreover, the clinical benefits of the removal of coccyx till need to be verified by robust evidence. There may be a suspicion of overtreatment in some patients. Fourth, pelvic reconstruction in ELAPE is challenging and needs assistance from a plastic surgeon [9]. In addition, the pelvic peritoneum is difficult to close in the prone jackknife position, which may lead to the descent of the small bowel into the pelvic dead space, increasing the risk of perineal complications [18].

In consideration of the aforementioned controversy and disadvantages regarding the patient’s position change in ELAPE, we modified the position change sequence and simplified the procedure. In this single-center, retrospective study, we introduce a modified position change of laparoscopic ELAPE and evaluate its feasibility, safety and the long-term therapeutic outcomes.

## Methods

### Patients

The clinical datas of consecutive patients with low rectal cancer (≤ 5 cm from the anal verge) underwent laparoscopic ELAPE procedure at the Department of Gastrointestinal Surgery of our hospital from November 2013 to September 2016 were retrospectively collected. The diagnosis of low rectal cancer was made by clinical finding, imaging examination and colonoscopy with biopsy. The preoperative staging of each patient was accurately assessed according to physical examination, Computed tomography (CT), colonoscopy and magnetic resonance imaging (MRI). All patients included in our study were in cT1N0M0-cT4N2M0 stage, and patients with cT3N + M0-cT4N2M0 underwent neoadjuvant chemoradiotherapy (nCRT). Preoperative defecation function and the risk of anastomotic leakage were strictly evaluated. The preoperative defecation function was clinically evaluated using the rating criteria of Williams et al. [19] Due to the suspicious circumferential resection margin involvement, poor preoperative defecation function and the high risk of anastomotic leakage, the patients with tumors more than 3 cm from the anal verge in this study were performed with ELAPE procedure, rather than traditional APE, transanal total mesorectal excision (TaTME), intersphincteric resection (ISR) and so on. Patient 15 had lesions invading to vagina, which was confirmed in surgery and by pathological examination. ELAPE procedure was not recommended for patient 55 with a cT1, and he had defecatory dysfunction. As the history of familial cancer and multiple first-degree relatives had died from rectal carcinoma, patient 55 felt extreme panic or fear. Despite multiple attempts to explain it, he was still strongly desired the ELAPE procedure. Thereby, we finally performed the ELAPE procedure for him. All procedures were performed by the same group of colorectal surgeons at the Department of Gastrointestinal Surgery in our hospital. The Medical Ethics Committee of our hospital approved the study protocol. Written informed consents were obtained from all the patients.

### Surgical technique

#### Perineal procedure

**Fig. 1:**
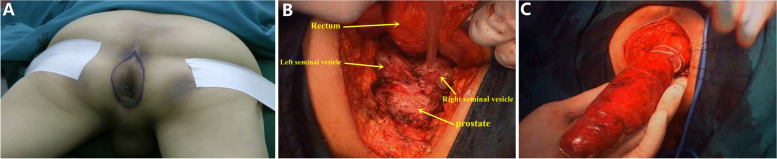
Perineal operative procedure. **A** incision range in the prone jackknife position, **B** rectum was separated to the upper rim of seminal vesicle in male patients, **C** the loop ligature of anus rectum was performed with a sterile specimen bag

The patient was in a prone jackknife position. The anus was closed by double purse-string sutures. Centered around the anus, an elliptical incision was made about 3 cm away from the anus, with the above to the apex of coccyx, the below to the midpoint of the perineum, and the side to the inside edge of ischial tuberosity (Fig. 1A). After incision of skins, the subcutaneous tissue was cut layer-by-layer with electricity knife. The surgeon separated the tissue space carefully along the ischial tuberosity and the inside edge of glutes fascia, and meanwhile resected the ischiorectal fossa fat tissue. The initiation of the levator ani muscle on both sides were then exposed and cut. Anococcygeal ligament was cut off in front of the apex of coccyx. The rear separation was along the anterior sacral fascia upper to 2–3 sacral vertebral plane. The front separation was along the perineal superficial temporal muscle trailing edge. The recto-urethralis and puborectal muscle were cut off and separated from posterior wall of urethra along the anterior wall of anorectum. In the plane of Denonviller’s Fascia, rectum was separated from tissue spaces of urethra, prostate and seminal vesicle to the upper rim of seminal vesicle in male patients (Fig. 1B). While in female patients, the front wall was separated along tissue spaces of rectum and vagina to peritoneal reflection. The lateral rectal ligaments were cut off. The rectum was then completely dissociated. In this procedure, the surgeon was required to pay attention to protecting the seminal vesicle gland. The pelvic autonomic nerves should be protected carefully during this procedure. The loop ligature of anus rectum was performed with a sterile specimen bag, and then it was sent to the pelvic cavity (Fig. 1C). After a sacroanterior drainage tube was placed, the perineum incision was sutured by a two-layer method involving skin and deep fascia. In addition, the pelvic peritoneum should not be opened for it may influence the visual field of abdominal operation.

#### Position changing process

After the perineal dissection in prone jackknife position, the patient’s position was changed to supine position. In our clinical practice, a set of efficient and safe position changing process was gradually established. A flatcar was first placed in parallel with the operating table. Two medical workers then stood at the side of the flatcar and another two stood at the side of the operating table. One medical worker at the flatcar side placed his hands on the shoulders and back of the patient, respectively, and another one put his hands on the hips and lower extremities of the patient, respectively. Then the other two medical workers in the opposite side turned over the patient to the upper limbs of two medical workers at the flatcar side and slowly placed the patient on the flatcar. Afterwards, the patient was moved to the operating table and placed in supine position. In the whole process of position change, an anesthetist controlled the patient’s head position to avoid hazardous situations such as cervical dislocation, trachea cannula exodus, and so on.

#### Laparoscopic procedure

**Fig. 2:**
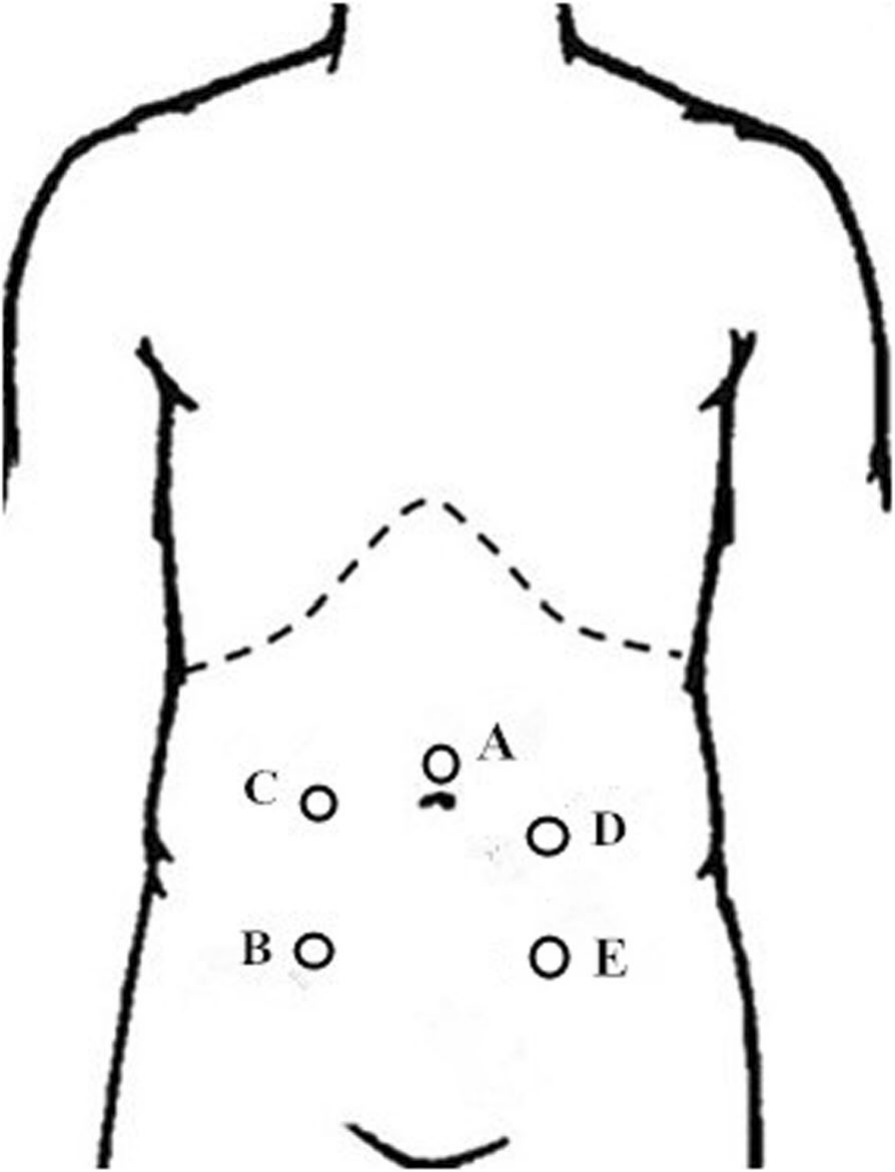
The location of the trocars (trocar **A** 10 mm, trocar **B** 12 mm, trocar **C**, **D** and **E** 5 mm)

The surgeon stood at the patient’s right side, the camera holder at the surgeon’s left side, and the first assistant at the patient’s left side. The monitor was placed at the patient’s feet side. The observation port was located above the umbilicus. The trocars of various size were placed as shown in Fig. 2. The trocar A (10 mm) was inserted through the observation port, then the pneumoperitoneum was created with a pressure of 13 mmHg. The trocar B (12 mm) which was mainly operated by the surgeon was placed at the intersection of the right midclavicular line and the anterior superior spine. The trocar C (5 mm) which was operated by the surgeon as an auxiliary was placed at the intersection of the right midclavicular line and the umbilicus. The trocars D and E (both were 5 mm) were used by the first assistant, and the trocar D was placed at the location of colostomy.

**Fig. 3:**
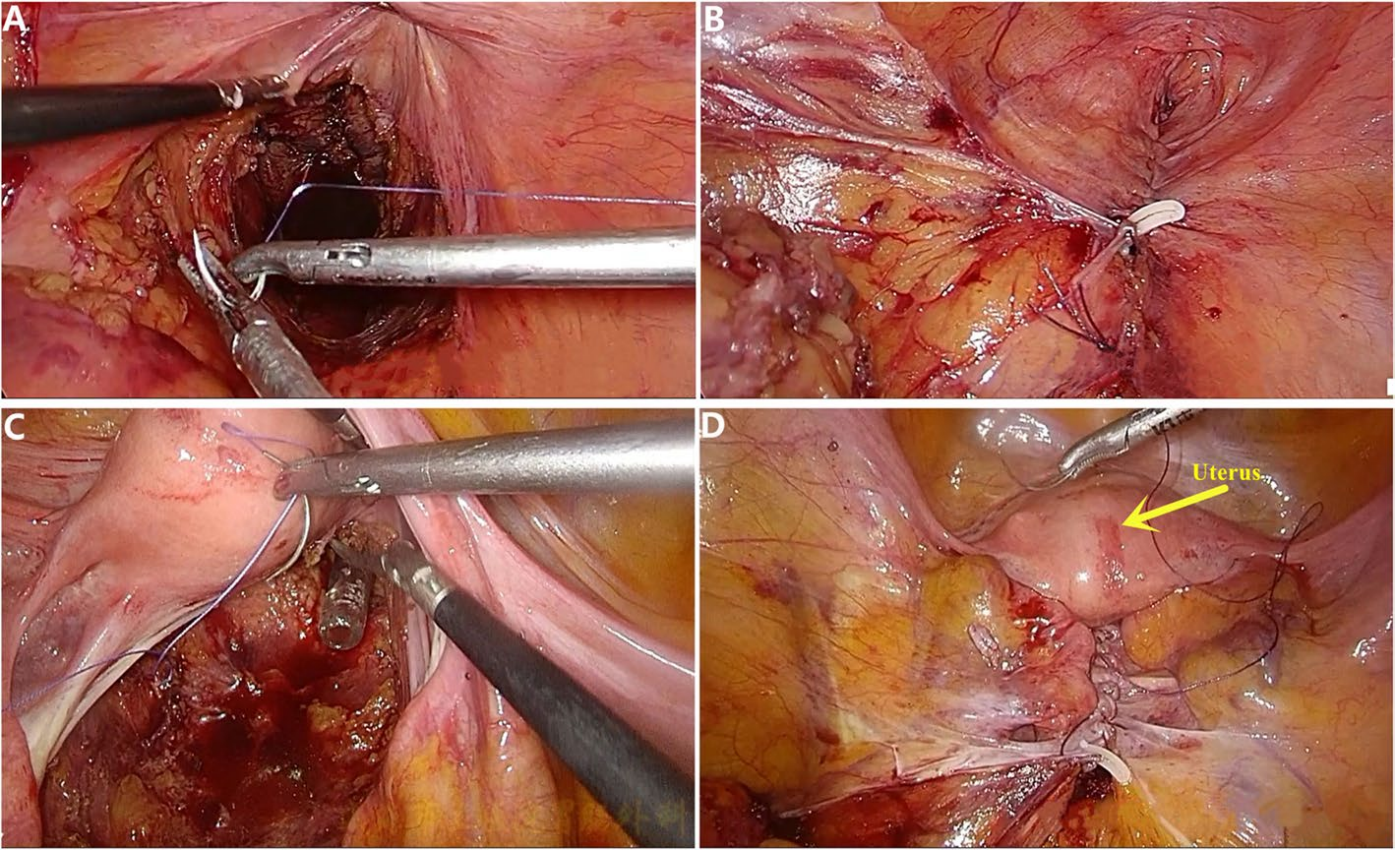
The pelvic peritoneum was directly closed by the laparoscopic approach. **A, B** the closure of pelvic peritoneum in the male patient; **C, D** the closure of pelvic peritoneum in the female patient

After inserting the laparoscopic devices into the abdomen, the patient was adjusted to the Trendelenburg position rightward. The sigmoid mesocolon was lifted up and the serosa at its root was sharply dissected by ultrasound knife. Enough residual serosal tissue should be retained to close the pelvic floor afterwards. Inferior mesenteric vessels were ligated and cut at its root through blunt and sharp dissections. No.253 lymph node was resected while protecting both sides of the waist perineal nerve and left colic artery. The left colic artery was ligated and cut at its remote branch. The dissection was carefully operated in Toldt’s space, and the ureter and superior hypogastric plexus were protected during this process. The rectum was isolated via sharp dissection of the posterior rectal wall from the retrorectal space and the anterior rectal wall from the posterior lobe of Denonvilliers fascia. The rectal was fully exposed and released until meeting with the perineal group. The total mesorectal excision with high vascular ligation was similar to that Samalavicius et al. [20] After the rectal specimen was removed from the minor pelvis, the pelvic cavity was washed with distilled water. Three to zero absorbable suture was used to continuously suture the serous layer of the pelvic wall to close the pelvic cavity and reconstruct the pelvic peritoneum (Fig. 3).

After the pneumoperitoneum, an incision in diameter of 3.5 cm was made at the location of the colostomy. The rectum and sigmoid colon were removed from the abdominal cavity after layer-by-layer incision of the skin and subcutaneous tissues. The sigmoid colon was transected at about 20 cm away from the border of the tumor. The specimen was then removed from the location of the colostomy and sent for pathologic examination. Afterwards, the colostomy was performed at the patient’s left lower abdomen.

### Follow-up

The patients were followed up every 3 months within 3 years post-operation, every 6 months at 4–5 years post-operation, and annually, thereafter, from the date of surgery till death or the cut-off date of September 15, 2021. Disease history inquiry, physical examination, hepatic ultrasonography scanning, serum CEA and CA199 were performed at each follow-up. Contrast-enhanced CT scanning was performed every 6 months post-operation. Colonoscopy was performed each year postoperatively. For male patients, sexual function was recorded through telephone follow-up.

### Statistical analysis

Data analyses were performed with IBM SPSS Statistics 26.0. Survival analysis was conducted by Kaplan-Meier method.

## Results

### Patient characteristics

A total of 56 consecutive patients (37 males, 19 females) with low rectal cancer underwent laparoscopic ELAPE from November 2013 to September 2016 and were included in our study. Patient characteristics are shown in Table 1. The mean age was 64.2 years (range 35–83), and mean BMI was 23.6 kg/m^2^ (range 17.3–34.3). The mean distance from tumor to anal verge was 2.9 ± 1.0 cm, and mean tumor size was 4 cm (range 1–8). Thirteen patients (23.2%) received nCRT, while the other patients such as patient 12, 29, and 36 refused preoperative treatment due to personal reasons. The preoperative MRI of patient 15 showed that the tumor invaded the vaginal wall, a situation in which nCRT was usually recommended. However, this patient did not receive nCRT because of tumor bleeding.

**Table 1:** Patient characteristics

Patient number	Gender	Age (years)	BMI (kg/m^2^)	ASA class	Distance to anal verge (cm)	Tumor size (cm)	Preoperative stage	nCRT
1	Male	68	22.7	2	3	3	cT2N1M0	No
2	Female	49	21.5	1	3	3.8	cT3N0M0	No
3	Male	63	23.5	1	4	4	cT3N2M0	Yes
4	Male	62	23.1	1	4	4	cT3N2M0	Yes
5	Female	69	25.4	2	3	3	cT3N0M0	No
6	Female	77	25.4	1	3.5	4.8	cT3N0M0	No
7	Male	56	25.4	1	2	4	cT3N0M0	No
8	Male	66	23.4	2	3.5	3.5	cT3N0M0	No
9	Male	59	21.5	2	3	6	cT3N0M0	No
10	Male	57	34.3	2	3	4.5	cT3N2M0	Yes
11	Male	68	19.1	2	3	2	cT2N0M0	No
12	Male	76	22.0	2	2	2.5	cT3N2M0	No
13	Male	68	23.6	2	3	3.5	cT3N2M0	No
14	Female	77	22.2	2	1	5	cT3N2M0	No
15	Female	66	27.6	2	4	5	cT4bN1M0	No
16	Male	58	25.9	1	4	3	cT3N1M0	No
17	Male	56	21.3	1	1	4.5	cT3N2M0	No
18	Female	72	22.1	2	3	5.5	cT3N0M0	No
19	Female	52	23.4	1	3	2.5	cT3N2M0	No
20	Male	66	23.7	1	3	3.5	cT2N1M0	No
21	Female	66	27.1	2	1	1.5	cT2N1M0	No
22	Female	57	21.3	2	3	3.5	cT2N1M0	No
23	Male	54	26.7	1	3	3	cT3N2M0	Yes
24	Male	49	22.6	1	4	6	cT3N0M0	No
25	Male	73	21.2	2	4	5	cT4N2M0	Yes
26	Male	40	23.7	1	5	4.8	cT4N2M0	Yes
27	Male	69	20.3	2	3.5	4.5	cT3N2M0	Yes
28	Female	72	23.2	2	1	7	cT2N0M0	No
29	Male	66	26.3	1	2	8	cT3N2M0	No
30	Male	65	23.0	1	4	4	cT3N0M0	No
31	Female	69	24.0	3	3	4	cT3N0M0	No
32	Female	75	22.4	1	4	6.2	cT4N2M0	Yes
33	Male	78	20.6	2	2	3.7	cT2N2M0	No
34	Female	66	23.2	2	4	3.8	cT3N0M0	No
35	Male	75	23.5	2	1	3	cT3N0M0	No
36	Male	57	20.8	2	5	3.8	cT3N2M0	No
37	Male	63	22.2	1	3	3.6	cT2N0M0	No
38	Male	35	22.5	1	3	5	cT2N0M0	No
39	Female	55	22.5	1	3	3.5	cT3N2M0	Yes
40	Male	47	21.3	1	4	2.5	cT2N0M0	No
41	Female	67	21.4	2	3	2.6	cT3N0M0	No
42	Male	58	27.7	2	4	3.5	cT3N0M0	No
43	Male	62	22.9	1	1	3.5	cT3N0M0	No
44	Male	58	17.3	1	1	7	cT3N0M0	No
45	Male	83	19.6	2	2	2.7	cT2N0M0	No
46	Male	69	25.1	2	2	5.5	cT2N0M0	No
47	Male	66	21.8	2	3	3	cT3N2M0	Yes
48	Male	58	23.0	1	2	2	cT2N2M0	Yes
49	Female	69	26.1	2	2	5	cT2N0M0	No
50	Male	56	26.1	1	3	7.3	cT2N1M0	No
51	Male	67	29.4	1	3	1	cT2N1M0	No
52	Female	60	26.7	2	2	3	cT3N0M0	No
53	Male	59	26.0	1	4	5	cT3N2M0	Yes
54	Male	69	23.9	1	3	3	cT3N1M0	Yes
55	Male	64	23.7	2	3	1.7	cT1N0M0	No
56	Female	71	22.9	1	4	4.5	cT3N0M0	No

*ASA* American society of anesthesiologists, *BMI* body mass index, *nCRT* neoadjuvant chemoradiotherapy

### Intraoperative and postoperative outcomes

Laparoscopic ELAPE was successfully performed in all the patients, without bowel perforation, conversion to open surgery and postoperative 30-day death. No patient underwent coccygectomy. The pelvic peritoneum was reconstructed in all the patients without the application of biological mesh. All the specimens were columnar and were mainly composed of anal canal, the middle and lower parts of the rectum, most of the levator ani muscle and mesorectum. The intraoperative and postoperative outcomes are summarized in Table 2. The mean operative time was 213.5 ± 29.4 min, and the mean amount of intraoperative blood loss was 152.7 ± 125.2 ml (range 50–800). Only patient 23 required intraoperative blood transfusion. The mean duration of postoperative hospital stay was 10.5 ± 1.8 days (range 7–13). The mean durations from surgery to urinary catheter removal was 4.5 ± 1.5 days (range 2–6), to first flatus was 2.4 ± 0.6 days (range 1–3), and to 1st liquid diet was 1.9 ± 0.7 days (range 1–4). The perineum incision healed well in all cases. No complications, in particular perineal complications, within 30 days post-operation were observed. During the long-term follow-up period, perineal hernia was observed only in patient 52 6 months after the procedure. There were 3 patients presented with parastomal hernias, which occurred at postoperative 17 months, 19 months, and 4 years, respectively. One patient presented with decreased sexual function. Other complications such as urinary dysfunction and sacrococcygeal pain did not occur in any patient.

**Table 2:** Intraoperative and postoperative outcomes

Patient number	Operative time (min)	Blood loss (ml)	Postope-rative hospital stay (days)	Days to first flatus	Days to urinary catheter removal	Days to 1st liquid diet	postoperative 30-day complications	Postoperative 30-day death	long-term complications
1	210	200	12	2	6	3	No	No	No
2	200	100	9	3	4	4	No	No	No
3	220	100	12	3	6	2	No	No	No
4	210	300	12	3	6	2	No	No	No
5	240	200	12	2	6	2	No	No	Parastomal hernia
6	240	200	12	3	6	3	No	No	No
7	200	200	8	2	6	1	No	No	Decreased sexual function
8	230	300	8	2	6	2	No	No	No
9	190	400	9	2	6	2	No	No	No
10	320	400	12	3	5	2	No	No	parastomal hernia
11	230	100	11	3	5	2	No	No	No
12	240	300	13	3	6	2	No	No	No
13	240	100	9	3	5	2	No	No	No
14	220	200	11	2	7	2	No	No	No
15	280	200	12	2	6	2	No	No	No
16	220	100	12	2	5	2	No	No	No
17	240	200	11	3	6	2	No	No	No
18	220	200	10	2	4	3	No	No	N0
19	210	200	8	2	5	2	No	No	No
20	200	200	9	3	5	4	No	No	No
21	245	50	12	2	5	2	No	No	parastomal hernia
22	200	200	9	3	5	2	No	No	No
23	240	800	9	3	4	2	No	No	No
24	205	100	8	2	3	2	No	No	No
25	240	100	12	3	6	2	No	No	No
26	230	200	13	3	4	3	No	No	No
27	240	200	13	2	6	2	No	No	No
28	205	200	9	2	5	2	No	No	No
29	210	300	13	2	3	1	No	No	No
30	190	100	11	3	5	2	No	No	No
31	210	100	12	2	5	2	No	No	No
32	200	200	11	3	4	1	No	No	No
33	230	50	10	3	3	2	No	No	No
34	220	100	11	2	6	2	No	No	No
35	190	100	11	2	6	2	No	No	No
36	170	50	8	2	6	1	No	No	No
37	160	100	12	2	4	1	No	No	No
38	230	100	8	2	3	1	No	No	No
39	230	50	11	1	5	1	No	No	No
40	170	50	11	2	6	3	No	No	No
41	170	50	11	3	2	1	No	No	No
42	240	50	11	3	5	2	No	No	No
43	200	50	9	2	6	2	No	No	No
44	160	50	10	2	4	2	No	No	No
45	200	50	8	3	3	1	No	No	No
46	240	100	15	3	3	1	No	No	No
47	210	50	11	2	2	2	No	No	No
48	250	50	9	1	3	2	No	No	No
49	210	150	9	2	2	1	No	No	No
50	180	100	11	2	2	1	No	No	No
51	180	100	9	2	2	2	No	No	No
52	170	100	11	2	2	1	No	No	Perineal hernia
53	190	100	7	2	4	1	No	No	No
54	200	100	11	3	2	1	No	No	No
55	200	50	13	3	2	1	No	No	No
56	180	50	8	3	3	2	No	No	No

### Pathologic and 5-year oncologic outcomes

**Fig. 4:**
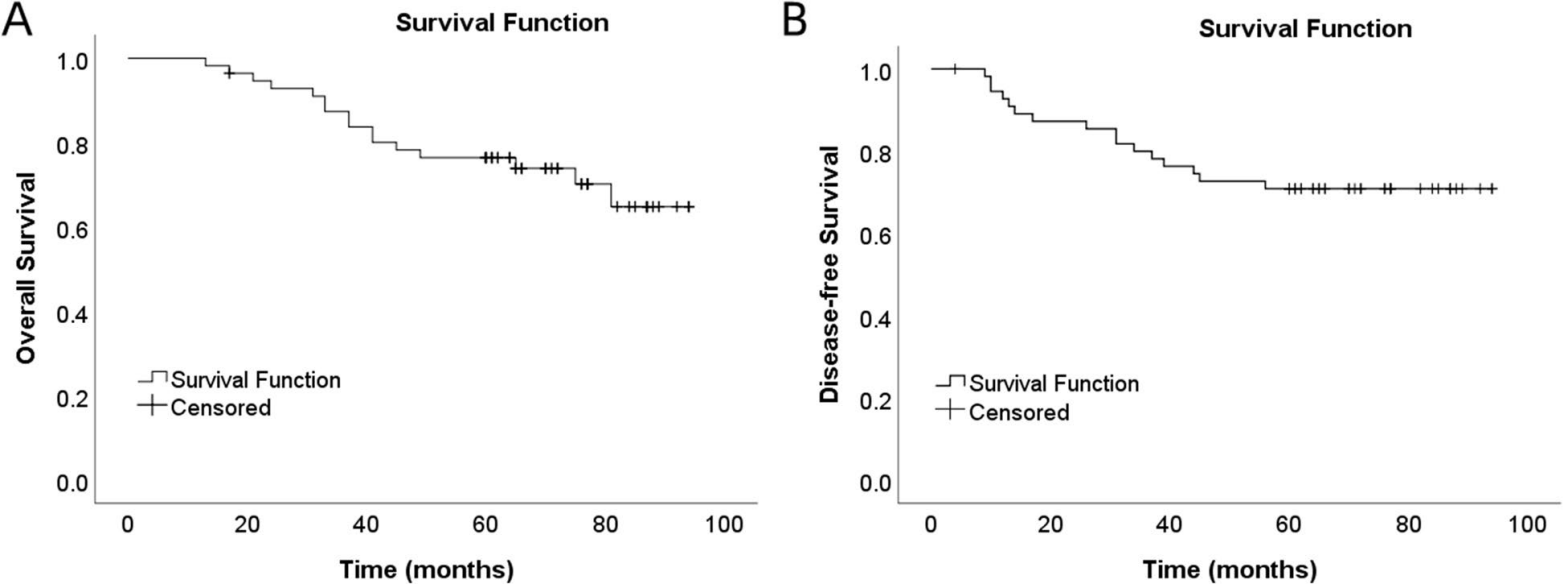
Kaplan-Meier curves showing overall and disease-free survival. **A** survival curves of overall survival. **B** survival curves of disease-free survival

All the tumors in the patients were completely resected, with negative CRM, upper and lower margins. Pathologic and 5-year oncologic outcomes are summarized in Table 3. Based on pathological TNM staging, there were 18 patients (32.1%), 21 patients (37.5%), and 17patients (30.4%) at stage I, stage II and stage III, respectively. According to the tumor cell differentiation degree, 6 patients (10.7%) had well-differentiated adenocarcinoma, 38 patients (67.9%) had moderately differentiated adenocarcinoma, and 12 patients (21.4%) had poorly differentiated adenocarcinoma. Five patients (8.9%) had mucinous adenocarcinoma, while 12 patients (8.9%) had venous or neural invasion. The median follow-up period was 65 months (range 13–94), only patient 48 lost to follow-up 17 months after surgery. During the follow-up period, only one patient (1.9%) suffered from local recurrence at 37 months post-operation, and the relapsed lesion was treated by surgical resection. Distant metastases occurred in 12 patients, of which 5 patients (41.7%) developed lung metastases, which is the most frequently observed distant metastasis type in low rectal cancer. There were 2 patients with bone metastasis, 1 with liver metastasis, 2 with lung metastasis accompanied by bone metastasis, 1 with lung metastasis accompanied by liver metastasis and 1 with systemic metastasis. Up to the cut-off date of follow-up, 16 patients (28.6%) died. Among them, 4 died from non-cancer related causes. The 3-year and 5-year overall survival (OS) rates were 87.3 and 76.4%, respectively. The 3-year and 5-year disease-free survival (DFS) rates were 80 and 70.9%, respectively (Fig. 4).

**Table 3:** Pathologic and 5-year oncologic outcomes

Patient number	Pathological stage	CRM	Differentiation degree	MAC	Venous or neural invasion	Local recurrence	Distant metastasis	Survival status
1	pT2N0M0 I	Negative	Well	No	No	No	No	Normal
2	pT3N0M0 IIA	Negative	Moderately	No	No	No	No	Normal
3	ypT3N1bM0 IIIB	Negative	Moderately	No	No	No	Lung	Death
4	ypT3N1aM0 IIIB	Negative	Poorly	No	No	No	No	Normal
5	pT3N0M0 IIA	Negative	Moderately	No	No	No	No	Normal
6	pT3N0M0 IIA	Negative	Well	No	No	No	No	Normal
7	pT3N0M0 IIA	Negative	Moderately	No	No	No	No	Normal
8	pT2N0M0 I	Negative	Well	No	No	No	No	Normal
9	pT3N0M0 IIA	Negative	Moderately	No	No	No	No	Normal
10	ypT3N2aM0 IIIB	Negative	Moderately	Yes	No	No	Lung	Death
11	pT1N0M0 I	Negative	Moderately	No	No	No	No	Normal
12	pT3N1bM0 IIIB	Negative	Poorly	No	Yes	No	Lung	Death
13	pT3N1aM0 IIIB	Negative	Moderately	No	Yes	No	No	Normal
14	pT3N0M0 IIA	Negative	Moderately	No	Yes	No	Lung	Death
15	pT4bN0M0 IIC	Negative	Moderately	No	No	No	No	Normal
16	pT3N0M0 IIA	Negative	Well	No	No	No	Lung and bone	Death
17	pT3N0M0 IIA	Negative	Moderately	Yes	No	No	No	Normal
18	pT2N0M0 I	Negative	Well	Yes	No	No	No	Death
19	pT2N0M0 I	Negative	Poorly	No	No	No	No	Normal
20	pT2N0M0 I	Negative	Moderately	No	No	No	No	Normal
21	pT1N0M0 I	Negative	Moderately	No	No	No	No	Normal
22	pT3N0M0 IIA	Negative	Moderately	No	No	No	No	Normal
23	ypT3N2aM0 IIIB	Negative	Poorly	No	Yes	No	No	Normal
24	pT3N0M0 IIA	Negative	Moderately	No	No	No	No	Normal
25	ypT3N2bM0 IIIC	Negative	Poorly	No	Yes	No	No	Death
26	ypT3N2bM0 IIIC	Negative	Poorly	No	Yes	No	Lung and bone	Death
27	ypT2N2aM0 IIIB	Negative	Moderately	No	No	No	Bone	Death
28	pT1N0M0 I	Negative	Moderately	No	No	No	No	Normal
29	pT3N0M0 IIA	Negative	Moderately	No	No	No	No	Normal
30	pT3N0M0 IIA	Negative	Moderately	No	No	No	No	Normal
31	pT3N0M0 IIA	Negative	Moderately	No	No	No	No	Normal
32	ypT3N2bM0 IIIC	Negative	Poorly	No	Yes	No	Lung	Death
33	pT2N1aM0 IIIA	Negative	Moderately	No	No	No	No	Normal
34	pT4aN0M0 IIB	Negative	Moderately	No	No	No	Lung, liver and bone	Death
35	pT2N0M0 I	Negative	Moderately	No	No	No	Bone	Death
36	pT3N2aM0 IIIB	Negative	Moderately	No	Yes	No	Liver	Death
37	pT2N0M0 I	Negative	Moderately	No	No	No	No	Normal
38	pT2N0M0 I	Negative	Poorly	No	No	No	No	Normal
39	ypT3N2aM0 IIIB	Negative	Poorly	No	Yes	No	No	Normal
40	pT2N0M0 I	Negative	Moderately	No	No	No	No	Normal
41	pT3N0M0 IIA	Negative	Poorly	No	No	No	No	Normal
42	pT2N0M0 I	Negative	Moderately	No	No	No	No	Normal
43	pT3N0M0 IIA	Negative	Moderately	No	Yes	Yes	No	Death
44	pT2N0M0 I	Negative	Moderately	No	Yes	No	No	Death
45	pT3N0M0 IIA	Negative	Moderately	No	No	No	Liver and lung	Death
46	pT1N0M0 I	Negative	Poorly	No	No	No	No	Normal
47	ypT3N1aM0 IIIB	Negative	Poorly	No	No	No	No	Normal
48	ypT3N2aM0 IIIB	Negative	Well	Yes	Yes	No	No	LTFU
49	pT2N0M0 I	Negative	Moderately	No	No	No	No	Normal
50	pT3N0M0 IIA	Negative	Moderately	Yes	No	No	No	Normal
51	pT2N0M0 I	Negative	Moderately	No	No	No	No	Normal
52	pT3N0M0 IIA	Negative	Moderately	No	No	No	No	Normal
53	ypT3N1aM0 IIIB	Negative	Moderately	No	No	No	No	Normal
54	ypT3N1aM0 IIIB	Negative	Moderately	No	No	No	No	Normal
55	pT1N0M0 I	Negative	Moderately	No	No	No	No	Normal
56	pT3N0M0 IIA	Negative	Moderately	No	No	No	No	Normal

*CRM* circumferential resection margin, *MAC* mucinous adenocarcinoma, *LTFU* lost to follow-up

## Discussion

In our modified ELAPE, the perineal procedure in prone jackknife position was firstly performed, and the abdominal procedure in supine position was then conducted. In the perineal operation, the coccyx was not routinely surgically removed, and the pelvic peritoneum was directly closed by laparoscopic approach without the application of biological mesh. The modified ELAPE was successfully performed in all the patients in this study, and this novel surgical technique was demonstrated to be safe, even in patients with advanced age (range 75–83 years). In this novel ELAPE, the sequence of perineal operation first and abdominal operation later avoids the squeeze of abdominal incision and colostomy stoma, and thus lowers postoperative complications associated with colostomy stoma, such as necrosis, stenosis, and parastomal hernia [17]. Moreover, the operative field in the perineal operation would not be influenced by the errhysis following the abdominal operation as seen in Holm’s ELAPE, which is critical to avoid pelvic vascular and nerval injury.

In our opinion, prone jackknife position provides a better surgical field to the surgeon, which enables a comfortable manipulation. In addition, the improved visual field helps the surgeon to clearly define the resection range, which in consequence reduce the incidence of intestinal perforation. Meanwhile, vascular and genital nerval injury could be avoided and thus the incidence of intraoperative bleeding and sexual dysfunction could be reduced. Research results by Dalton et al. [16] and Shiha et al. [21] suggested that the prone jackknife position in perineal operation in ELAPE was more suitable for patients with tumor infiltrating the prostate and posterior vaginal wall. No positive CRM and intraoperative perforation were found in our study, and only 1 male patient developed sexual dysfunction, the incidence of which was lower than that reported previously [22]. In our experience, although the change of the patient’s position seemed to increase surgical risk and prolong the operative time, but the application of prone jack-knife position to the perineal operation was convenient for the surgeon to operate under the direct vision and clear surgical level, in addition, it could shorten the learning curve. Generally speaking, this reduced perineal complications and shortened the overall operation time instead. The mean operative time was 213.5 min, which was less than that reported in the previous studies [10, 23, 24]. With the accumulation of technical experience and improvement of laparoscopic techniques, the overall operation time of this novel ELAPE performed by our surgical team had been reduced to currently around or less than 180 minutes.

Perineal pain was the common postoperative complication in ELAPE. It has been indicated that the postoperative perineal pain may be related to the coccygectomy, the activation of inflammatory cytokines at the mesh site, the damage to the pudendal nerve, the wider excision of the levator ani muscles and ischiorectal fossa fat, and the suturing of the mesh itself close to the pelvic wall [22], among which the coccygectomy may be the main relation [25]. In ELAPE of Holm et al. [9], the coccyx is routinely resected to permit entry into the pelvic cavity at the point where the intra-abdominal dissection stopped, and the mesorectum needs to be turned out from the pelvis, followed by removal of the specimen from the perineal incision. Partial distal sacrum may even be resected in case the mesorectum is hypertrophy or the tumor is relatively huge. In our modified ELAPE, the specimen was removed from the location of colostomy, thus coccygectomy was not required in all cases. Whether to perform coccygectomy should be determined by the location of rectal tumor and the extent of invasion, the information of which was obtained by careful evaluation of the preoperatively MRI imaging. If the tumor locates at the anterior and lateral wall of the rectum, the coccyx could be retained. In case the tumor locates at the posterior wall of the rectum, the coccyx could still be retained in the condition of ensuring negative CRM. No patients in the present study underwent coccygectomy, and no sacrococcygeal pain occur in any patient.

The wider excision of the levator ani muscles and ischiorectal fossa fat leads to a large perineal defect at the level of the pelvic floor, which might result in increasing incidences of perineal complications, such as perineal wound infection and perineal hernia. The reconstruction of pelvic floor is critical for decreasing perineal morbidity. The currently reported methods of pelvic floor reconstruction mainly include primary closure [26], reconstruction with myocutaneous autologous flaps [27], reconstruction with biologic meshes [22, 25], and the pedicled omentoplasty [28]. Though the studies reported acceptable or favorable results regarding the methods mentioned above, no consensus was achieved on the optimal method. A recent multi-center retrospective study indicated that the application of biological mesh could not reduce the incidence of perineal hernia, and even increase perineal morbidity [29]. Result from a meta-analysis study found that compared to primary closure, reconstruction with biologic mesh was associated with a lower hernia rate, but it had no effect on perineal wound complications [30]. Therefore, the benefit of the application of biologic mesh in pelvic floor reconstruction remains controversial. In our opinion, wider excision of the ischiorectal fat is not necessary if the tumor do not infiltrate the ischiorectal fossa, and in this situation, the usage of gluteal muscle flap or biological mesh implants to pelvic floor reconstruction could be avoided. In all the cases in our study, the perineum incision was easily sutured in two layers and the pelvic peritoneum was closed laparoscopically, without coccygectomy and complex pelvic reconstruction. The closure of pelvic peritoneum has been reported to prevent the small bowel from descending into the pelvic cavity, thus avoiding perineal hernia and obstruction caused by adhesion of the small bowel in the pelvis [18]. In our study, no small-bowel obstruction or perineal wound infection was observed, and only 1 patient had perineal hernia during the long-term follow-up period. The low incidence of perineal complication in our study may not only be related to the closure of pelvic peritoneum, but be also associated with the retention of coccyx and the routine use of presacral drains.

Discrepancies in regard to the long-term outcomes of ELAPE can be found in previous studies. A retrospective study with long-term follow-up period showed that the local recurrence rate was 7% in ELAPE group, whereas long-term survival did not differ between ELAPE group and APE group [23]. Results from a recent single-center study revealed that the local recurrence rate of ELAPE reached 6.7%, and the 3-year and 5-year OS rates were 86.4 and 58.8%, respectively [31]. In the present study, the local recurrence rate of the modified ELAPE was 1.9% and the median observation time was 65 months. Pulmonary metastasis was the most frequently observed distant metastasis, followed by bone metastasis, and hepatic metastasis. This result was in consistent with a previous study conducted by Qiu et al. [32] The 5-year OS and DFS rates of this study were 76.4 and 70.9%, respectively. As is well known, ELAPE procedure is recommended for T4 tumors or advanced T3 tumors. Some patients with T1/2 tumors more than 3 cm from the anal verge in our consecutive series were performed with ELAPE procedure for reasons mentioned earlier. According to the pathologic and 5-year oncologic outcomes of these patients, we suggested that extended excision of the pelvic floor was not necessary in higher T1/2 tumors without infiltration of the pelvic floor or incontinence as indication. The main limitation of the present study is that it was a retrospective cohort study, which lacked control groups. Another limitation of this study is the small sample size with single institution.

## Conclusion

In the present study, with modified position change process and simplified procedure, laparoscopic ELAPE could be successfully completed, with favorable oncologic outcomes and low incidence of complications. This novel technique avoids the squeeze of the abdominal incision and colostomy stoma, and thus lowers postoperative complications associated with colostomy stoma. In addition, the pelvic peritoneum could be closed by laparoscopy without pelvic floor reconstruction using the gluteal muscle flap or biological mesh implants. In view of the limitation of the small sample size, further study with a large sample size is needed to confirm the feasibility of this modified surgical technique.

## Supplementary Information

**Additional file 1.**

## Data Availability

All data generated or analysed during this study are included in this published article and its Supplementary information files.

## Abbreviations

APE: Abdominoperineal excision
CRM: Circumferential resection margin
AR: Anterior resection
TME: Total mesorectal excision
ELAPE: Extralevator abdominoperineal excision
nCRT: Neoadjuvant chemoradiotherapy
MRI: Magnetic resonance imaging
OS: Overall survival
DFS: Disease-free survival
ASA: American Society of Anesthesiologists
BMI: Body mass index
MAC: Mucinous adenocarcinoma
LTFU: Lost to follow-up
